# Toxicomicrobiomics: The Human Microbiome vs. Pharmaceutical, Dietary, and Environmental Xenobiotics

**DOI:** 10.3389/fphar.2020.00390

**Published:** 2020-04-16

**Authors:** Nehal Adel Abdelsalam, Ahmed Tarek Ramadan, Marwa Tarek ElRakaiby, Ramy Karam Aziz

**Affiliations:** ^1^ The Center for Genome and Microbiome Research, Cairo University, Cairo, Egypt; ^2^ Department of Microbiology and Immunology, Faculty of Pharmacy, Cairo University, Cairo, Egypt

**Keywords:** microbiota, microbiome, pharmacomicrobiomics, metabolism, secondary metabolism, cytochromes

## Abstract

The harmful impact of xenobiotics on the environment and human health is being more widely recognized; yet, inter- and intraindividual genetic variations among humans modulate the extent of harm, mostly through modulating the outcome of xenobiotic metabolism and detoxification. As the Human Genome Project revealed that host genetic, epigenetic, and regulatory variations could not sufficiently explain the complexity of interindividual variability in xenobiotics metabolism, its sequel, the Human Microbiome Project, is investigating how this variability may be influenced by human-associated microbial communities. Xenobiotic-microbiome relationships are mutual and dynamic. Not only does the human microbiome have a direct metabolizing potential on xenobiotics, but it can also influence the expression of the host metabolizing genes and the activity of host enzymes. On the other hand, xenobiotics may alter the microbiome composition, leading to a state of dysbiosis, which is linked to multiple diseases and adverse health outcomes, including increased toxicity of some xenobiotics. Toxicomicrobiomics studies these mutual influences between the ever-changing microbiome cloud and xenobiotics of various origins, with emphasis on their fate and toxicity, as well the various classes of microbial xenobiotic-modifying enzymes. This review article discusses classic and recent findings in toxicomicrobiomics, with examples of interactions between gut, skin, urogenital, and oral microbiomes with pharmaceutical, food-derived, and environmental xenobiotics. The current state and future prospects of toxicomicrobiomic research are discussed, and the tools and strategies for performing such studies are thoroughly and critically compared.

## Xenobiotics and Their Metabolism

Xenobiotics are *chemical substances not normally present in the environment of living organisms.* They are mostly regarded as synthetic substances, but the term may be more loosely used to include naturally occurring chemicals and endobiotics, when present in higher concentrations than their normal levels, or produced by certain organisms as a defense mechanism, such as the toxins produced by some fungi, bacteria, or even herbs (Soucek, 2011). From a metabolism viewpoint, they can be defined as *chemicals that are extrinsic to the normal metabolism of a living organism* (Koppel et al., 2017). Food with its variety of dietary compounds, environmental chemicals and pollutants, as well as medications are considered xenobiotics to the human body (Koppel et al., 2017).

Xenobiotic metabolism increases their water solubility, thus enhancing their elimination from the body (Clarke et al., 2019). Oral ingestion of xenobiotics passes them to the upper gastrointestinal tract, and those absorbed are translocated to the liver through the hepatic portal vein, a process commonly known as the first pass effect. The human liver, using its cytochrome P450 (CYP 450) family of enzymes, chemically transforms both endogenous and exogenous compounds (Nelson, 2005; Michalopoulos, 2007). Liver metabolism has three phases (Soucek, 2011): phase I (activation by oxidation, reduction, or hydrolysis), phase II (conjugation to polar moieties), and phase III (transport without chemical modification). Xenobiotics that are not metabolized and excreted accumulate in the body and may lead to chronic diseases and inflammation (Jain et al., 2005).

Poorly absorbed xenobiotics pass from the small intestine to the large intestine, where they are exposed to the gut microbial metabolizing niche. Metabolites released in the circulation are either excreted by the kidneys or return through biliary duct to the gut. The final fate of these metabolites is either excretion in the stool or reabsorption in the small intestine (Koppel et al., 2017).

## Host Genetic Variability and Toxicogenomics

The efficiency of the host metabolizing enzymes varies from one individual to another according to the individual’s genetic makeup. Single nucleotide polymorphisms (SNPs) are among the reasons why individuals respond differently to xenobiotics and drugs (Soucek, 2011). Pharmacogenomics studies the impact of genetic discrepancies between individuals (usually SNPs) on their responses to drugs (Rizkallah et al., 2010; Saad et al., 2012). For example, genetic variants in uridine 5′-diphospho-glucuronosyltransferase (UGT) render some patients to slowly metabolize certain drugs such as NSAIDs and irinotecan due to lower glucuronidation rates (Lankisch et al., 2008; Stingl et al., 2014).

Likewise, toxicogenomics studies the impact of genetic discrepancies between individuals on their response to toxic substances. Philosophically speaking, toxicogenomics and pharmacogenomics are two manifestations of one phenomenon, given that all drugs can be regarded as poisons used at a non-toxic doses (Aziz, 2018). One may even go to an extreme and consider every chemical as a poison, following the classical principle of Paracelsus: *“Sola dosis facit venenum”* (Lt: only the dose makes the poison (quoted in Uppal et al., 2016)).

Although the host enzymes (notably liver enzymes) are variable and capable of metabolizing xenobiotics, their variability alone cannot explain the biotransformation of indigestible xenobiotics and dietary compounds. Human and microbial chemical transformations form a complex interchangeable network, in which they mutually affect one other (Soucek, 2011).

## The Microbiome

### The Human Microbiome: A Cloud Shrouded in Mystery

The microbiota is defined as all the microbial communities living on or in a particular biological system, formerly referred to as *flora* or *microflora*. The human body carries trillions of microbes with genes estimated to be at least 100 times more numerous than human coding genes. The term microbiome, disambiguated elsewhere (Saad et al., 2012), refers to a micro-ecosystem (micro.biome) or the summation of a microbiota and the genomes of its members (microbial metagenome). Sometimes it even describes the entire environment in which microbes reside (Marchesi and Ravel, 2015). Finally, *microbiomics* is defined as the field that uses “high throughput molecular techniques to study microbial communities” (Rajendhran and Gunasekaran, 2010).

A human’s microbiome, from one’s birth throughout his/her life, continuously evolves, impacting the body in both states of health and disease. Variations within a microbiome are not only developmental, but are also spatial, temporal, and are definitely affected by diet, hormones, stress, and even diurnal cycle. This continuous, multidimensional variability makes an individual’s microbiome akin to a cloud, with uncertainty of the exact composition and gene pool at any point of time and space (Elrakaiby et al., 2014).

### The Human Microbiome: A Brief History

After the Human Genome Project (HGP) has come to a conclusion in 2003, researchers were baffled by its unexpected results (Relman and Falkow, 2001). Coding genes in the human genome were found to be much fewer than previously expected. The genetic, epigenetic, and regulatory variations in the human genome were not sufﬁcient to explain the complexity of interindividual variability and its associated phenotypes. The human microbiome came then to the spotlight, with the promise that studying its composition, variability, and functional potential will allow the full understanding of the landscape of human phenotypic variations, and its effect on human health, immunity, and drug response.

In 2008, the NIH-funded Human Microbiome Project (HMP) was launched to explore intraindividual and interindividual variability influenced by the human microbiome (Turnbaugh et al., 2007). The project’s goals at launch were to (i) take advantage of the current high-throughput technologies to study the human microbiome by analyzing samples taken from multiple body sites (gastrointestinal tract, mouth, vagina, and skin) of more than 250 healthy volunteers; (ii) explore the microbiome’s role in health and disease by studying its variability under different medical conditions; (iii) construct a network that provides up-to-date data sources, new findings, standardized techniques, and technological approaches for the research community.

Currently, the HMP is in its second phase (the Integrative Human Microbiome Project or iHMP), characterized by focusing on selected health disorders (preterm birth, inflammatory bowel disease, and type 2 diabetes) to understand the microbiome’s role as well as the host involvement and interaction with its microbiome. With this second phase, a deluge of data, peer-reviewed articles, and news features with various degree of sophistication continue to tackle the role of the microbiome in almost every health condition.

In parallel to the rising excitement about microbiome research, which sometimes mixes established science with publicity, a handful of applications are revitalized, notably in the use of probiotics, functional foods, and nutraceuticals, and novel research areas are taking shape. Of these, two areas of research have expanded that relate to microbial interactions with chemicals to which the host is exposed: *pharmacomicrobiomics* is a relatively new field that investigates the interactions between human-associated microbes and drugs (Rizkallah et al., 2010) and *toxicomicrobiomics* is a branch of toxicology that extends toxicogenomics to exploring xenobiotic-microbiome interactions (Aziz et al., 2018).

### The Microbiome Cloud in Health and Disease

Early data collected from the HMP (Turnbaugh et al., 2007) showed that samples from different body sites (oral, skin, gastrointestinal tract, and urogenital tract) contained unique microbial communities in the same individual. Surprisingly, researchers also found that if samples were drawn from two different individuals, one individual’s oral sample will look more similar to the other’s oral sample than his or her own skin sample (Turnbaugh et al., 2007).

Unlike the human genome, the gut microbiome is far from stable all through human life. In fact it varies all the time as it responds to multiple factors, including diet and the exposure to different xenobiotics (Clarke et al., 2019).

From one’s birth throughout his/her adulthood, the human microbiome continues to shift and change in an endless cycle influenced by numerous factors. At birth, newborns were shown to have the same microbes across all of their body parts; however, based on their method of delivery, the first acquired microbes vary greatly (Dominguez-Bello et al., 2010). Babies born through vaginal delivery have *Lactobacillus* spp. as their first microbes, along with a typical profile of their gut microbiota and a proper breast milk digestion support. Meanwhile, babies born through C-section have *Staphylococcus* spp. as their first microbes, along with an abnormal gut microbiota and a predisposition to a variety of food allergies and other disorders (e.g., asthma, atopic diseases, allergic rhinitis, and celiac disease).

Studies have also shown that breastfeeding plays a vital role in developing normal microbiome. A study conducted on 107 mother-newborn pairs in 2017 found that 30% of beneficial bacteria in a newborn’s intestinal tract are acquired by seeding during breastfeeding (Pannaraj et al., 2017). A prior study in 2016 demonstrated that early antibiotic use in newborns resulted in the loss of microbial diversity and early overabundance of resistance genes in the microbiome (Langdon et al., 2016).

As healthy infants grow up, microbial communities become more diverse across different body parts, until they resemble those of an adult. However, an adult’s microbiome cloud continues to change because of other factors, such as age, diet, genetic makeup, environmental factors, physiological, and psychological changes. As a person grows older, the microbiome tends to be stable because of reduced frequency of activities and motility, lower exposure to environmental factors, chronic diseases, etc. (Spor et al., 2011). Both long-term (Wu et al., 2011) and extreme short-term (David et al., 2014) changes in dietary habits drastically change the gut microbiota. The gut-brain microbiome communication axis is affected by alteration in the psychological and physiological state of a person (Grenham et al., 2011).

Excessive use of antibiotics in adulthood was found to result in irreversible changes in the human microbiome (Dethlefsen and Relman, 2011). All of the previously mentioned factors along with many more (environmental factors, daily habits, hygiene, etc.) contribute to the loss of microbial diversity shifting the microbial cloud to a state of dysbiosis.

In a healthy body state, human-associated microbes carry their beneficial roles by protecting the body against exogenous microbes and triggering non-specific immune responses against them (protective effect), producing essential metabolites required by the human body (productive effect), and aiding in the digestion and metabolism of food and xenobiotics (digestive and metabolic effect). The microbiota is said to be in a state of ***symbiosis*** or ***eubiosis***. A diseased body state, on the other hand, is preceded by a shift towards microbial ***dysbiosis***. In dysbiosis, the balance between different microbes is disturbed. Often, commensal or opportunistic microbes convert to pathogenic ones *via* horizontal gene transfer (HGT) or a break in organ barriers causes bacteria to infect sterile body parts or opportunistic microbes taking advantage of immunocompromised patients to cause diseases (Petersen and Round, 2014).

Several studies, over the past decade, have described associations between microbiome variations and various adverse health outcomes. The microbiome has been linked to the development of a diverse set of diseases ranging from minor ailments to inflammatory and degenerative diseases (e.g., obesity (Maruvada et al., 2017), inflammatory bowel diseases (Kostic et al., 2014; Moustafa et al., 2018), stage 4 hepatitis C (Aly et al., 2016), and various cancers (Elinav et al., 2019). With any such reported association, a cause-and-effect question is raised: do microbiome variations play a role in disease causation or is it just an association that could merely be used as a biomarker for the adverse health outcome (Maruvada et al., 2017; Rosato et al., 2018). For example, causation has been demonstrated in inflammatory bowel diseases (Moustafa et al., 2018) and colorectal cancer (Tjalsma et al., 2012; Scott et al., 2019).

In either case (correlation or causation), microbiome-driven biomarkers are valuable diagnostics. In the case of mere correlation, these biomarkers would be signs of exposure to a certain adverse health outcome, while, when causation is established, microbiome biomarkers are of predictive value (e.g., *Fusobacterium nucleatum* (Kostic et al., 2013; Rubinstein et al., 2013) and signature microbial metabolites (Vipperla and O’keefe, 2016) as predictors of colorectal cancer).

### Microbiome-Driven Xenobiotic Metabolism

With its immense diversity, the human microbiome has a powerful metabolizing capacity that even exceeds its host’s metabolic potential. The human gut microbiome, in particular, is capable of the biotransformation of xenobiotics starting from dietary compounds to pharmaceutical ingredients. The microbiome can change xenobiotics half-lives, their potential biological effect on the human body, and the rate and extent by which they reach the blood circulation or their biological targets/receptors (Koppel et al., 2017). Whether a xenobiotic is poorly absorbed and passes through the host’s small into large intestine, binds to one of the efflux proteins, or whether it is absorbed into the circulation, it will be exposed at some stage to the microbiota and its enzymes (Hall et al., 1999). The less a xenobiotic is absorbed or the more it is bound to proteins, the longer it resides in the gut and the more it is exposed to variable microbial enzymes (Hall et al., 1999). Microbial enzymes can activate prodrugs, inactivate drugs or increase or alleviate toxicity of others (Spanogiannopoulos et al., 2016; Kuntz and Gilbert, 2017; Wilson and Nicholson, 2017). The gut microbiome may also indirectly influence the host’s capacity to metabolize xenobiotics or drugs (Koppel et al., 2017).

The metabolic enzymatic capacity of the gut microbiota is clearly distinguished from the host’s metabolic capabilities to a degree that the microbial metabolism of xenobiotics is sometimes opposite to the host biotransformation. Host enzymes mainly carry out oxidation and conjugation, while microbial enzymatic reactions are mainly reduction and hydrolysis (Spanogiannopoulos et al., 2016; Wilson and Nicholson, 2017). Demethylation is another example of discrepancies between host and microbiome xenobiotic metabolism: whereas the host demethylation renders a xenobiotic more polar to be excreted outside the body, for microorganisms, demethylation generates carbon sources for further growth and division (Sutton et al., 1997; Kumano et al., 2016).

The microbial enzymatic repertoire in the human gut is complex and consists of divergent enzyme types, such as beta-glucosidases, beta-glucuronidases, aryl sulfatases, azoreductases and nitroreductases (**Table 1**). The human microbiome contributes to interindividual variations, not only because of the diversity of microbial types within an individual, but also because microbial enzymes differ from one species to another in sequence, activity, and abundance (Spanogiannopoulos et al., 2016; Wilson and Nicholson, 2017).

**Table 1:** Some gut bacterial enzyme families encoded in the microbiome and their impact on xenobiotics.

Microbial Enzyme/Enzyme Family	Impact on Xenobiotics	Reference
Beta-glucosidases	Hydrolyze sugar moiety in plant glucosides	(Dabek et al., 2008)
Beta-glucuronidases	Hydrolyze glucuronic acid component from polysaccahrides and drug metabolites	(Pellock et al., 2019)
Sulfatases	Break sulfate esters of phase II host metabolism	(Ulmer et al., 2014)
Azoreductases	Reduce azodyes	(Misal and Gawai, 2018)
Nitroreductases	Less polar metabolites resulting in more toxic or inactive metabolites	(Swanson, 2015)
Proteases	Hydrolyze polypeptide chains	(Tozaki et al., 1997)
Glycosidases	Render molecules less polar	(Saitta et al., 2014)
C–S beta-lyases	Generate ammonia for bacterial growth	(Rossol and Puhler, 1992)
Transferases	Transfer methyl or acyl groups	(Koppel et al., 2017)

Hydrolysis, lyase-catalyzed bond breakage, reduction, and group transfer are some of the chemical reactions that gut microbial enzymes can catalyze (Koppel et al., 2017). Glycosidases, proteases, and sulfatases are among microbial hydrolytic enzymes. Glycosidases break the glycosidic bonds using water to release sugars (Kallemeijn et al., 2014). The xenobiotic molecule becomes less polar and thus acquires longer half-life in the human body which may increase it toxicity as seen in the non-steroidal anti-inflammatory drugs as diclofenac and ketoprofen (Saitta et al., 2014). Proteases break the bonds between amino acids in a polypeptide chain with different specificities (Tozaki et al., 1997). Phase II host metabolism results in sulfate ester which is broken down by sulfatases (Ulmer et al., 2014).

The bond cleavage, which neither requires oxygen nor water, is catalyzed by lyases. Microbial C–S beta-lyases break the carbon-sulfur bonds and the ammonia generated is the nutrient for bacterial growth (Rossol and Puhler, 1992). Reduction plays a role in opposing host metabolism mechanisms such as rendering xenobiotics less polar or drugs to either their inactive or active forms (Peppercorn and Goldman, 1972; Lavrijsen et al., 1995; Haiser et al., 2013). Methyl or acyl groups are transferred from or to xenobiotics by microbial transferase enzymes (Koppel et al., 2017). Acylation of 5-aminosalicylic acid, for example, leads to inactivation of the drug (Delomenie et al., 2001).

The presence or absence of a gut microbiota can have an indirect effect on regulating host metabolizing enzymes, either through competition of the microbial metabolites on host enzymes or through changing the expression of these enzymes. When gnotobiotic mice were studied in comparison to regular mice, the expression of liver enzyme genes, including active androstane receptor, was upregulated in germ-free mice (Bjorkholm et al., 2009). Expression levels of CYP3A (cytochrome P450 family 3 subfamily A) in mice decreased upon administration of ciprofloxacin antibiotic. This downregulation was attributed to the gut bacteria synthesizing lithocholic acid, which was hypothesized to affect CYP expression (Toda et al., 2009). In germ-free mice, transcription and translation of CYP3a11, the mouse homolog of human CYP3A4, were significantly lessened. However, gene expression of both CYP1a2 and CYP4a14 was elevated in germ-free mice (Selwyn et al., 2016).

Elemicin is an essential oil constituent incorporated into food and dietary supplements, which upon activation by CYP1A2 (the human homolog of mouse CYP1a2), is converted to more potential cytotoxic metabolite (Wang et al., 2019). CYP1A2 affects potent medications as theophylline, psychological disorders medications, warfarin, and other anticancer drugs (Wishart et al., 2018). CYP4a14 is the homolog of human CYP4A which is mainly responsible for arachidonic acid and fatty acids metabolism and may play a crucial role in prognosis of nonalcoholic fatty liver disease (Zhang et al., 2017b). Gene expression regulation of some of the host detoxifying enzymes such as glutathione peroxidases, sulfotransferase, epoxide hydrolases, and N-acteyltransferases was shown to be affected by the absence of gut bacteria (Meinl et al., 2009).

Gut microbiota-derived metabolites of steroid hormones, uremic solutes of dietary protein breakdown or herbal medicines, or bile acids may decrease CYP3A4 expression (Bjorkholm et al., 2009; Devlin et al., 2016). Low CYP3A4 results in less clearance of multiple CYP3A4-dependant medications, e.g., erythromycin and verapamil, affecting their action and adverse effects (Barnes et al., 2014).

### Pharmaceutical Xenobiotics

The use of pharmaceutical products is undoubtedly on the rise over the past decades. According to the Global Use of Medicines report from the IQVIA Institute for human data science, the global market for pharmaceuticals reached $1.2 trillion in 2018, up $100 billion from 2017 (The Iqvia Institute, 2019). With the availability of pharmacies around every corner offering over-the-counter pharmaceutical products and the increasing number of patients with chronic illnesses, using such products becomes an integral part of our everyday lives. Effects of pharmaceuticals on the human body have long been studied, but with the importance of the microbiome lately being rediscovered, study results are accruing on the mutual interplay between pharmaceuticals and the microbiome.

As previously established, different body sites (oral, skin, gastrointestinal tract and urogenital tract) contain unique microbial communities (Turnbaugh et al., 2007), each of which interacts with pharmaceuticals in a different way (affecting and being affected by them). Some interactions may lead to an increase or a decrease in activity, while others result in increased toxicity of a pharmaceutical xenobiotic (**Table 2**), which fall within the scope of toxicomicrobiomics.

**Table 2:** Adverse effects/toxicities resulting from xenobiotic-microbiome interactions (pharmaceutical toxicomicrobiomic interactions).

Xenobiotic	Microbes	Effect	Reference
Carboplatin	*Prevotella copri*	In a rat model, carboplatin-induced intestinal mucositis was reduced after metronidazole treatment, which was intended to target the anaerobic *P. copri*.	(Yu et al., 2019)
Doxorubicin	Intestinal microbiota	The enteric microbiota was found to play a major role in doxorubicin-induced mucositis. Targeting the microbiota reduced damage.	(Rigby et al., 2016)
Irinotecan	Intestinal microbiota	Deconjugation of SN-38G (Irinotecan pharmacologically active derivative) in the gut by bacterial beta-glucuronidases resulted in gut toxicity.	(Takasuna et al., 1996)
5-Fluorouracil	Intestinal microbiota	Treatment with 5-FU was shown to cause significant changes in intestinal microbiota and mucin secretion in a rat model. These changes contributed to development of 5-FU-induced mucositis.	(Stringer et al., 2009)
Acetaminophen	Intestinal microbiota	Elevated levels of p-cresol (produced by some intestinal bacterial communities, which are not equally abundant in all individuals) compete with acetaminophen on the o-sulfonation metabolism, which results in increased acetaminophen toxicity.	(Clayton et al., 2009)
Sorivudine	Bacteroides	Sorivudine hydrolysis by intestinal anaerobic bacteria results in high blood concentration of 5-(E)-(2-bromovinyl)uracil increasing the level and toxicity of 5-fluorouracil during chemotherapy.	(Nakayama et al., 1997)
Cycasin	Intestinal microbiota	Cycasin hydrolysis by the gut microbiota resulted in a hepatotoxic and carcinogenic methylazoxymethanol.	(Spatz et al., 1967)
Indomethacin	Luminal bacteria	Interaction of the oral NSAID, indomethacin, with gut microbes resulted in damage to the intestinal mucosa.	(Basivireddy et al., 2005)
Nitrazepam	*Clostridium leptum*	Nitrazepam reduction by the clostridial nitroreductase enzyme was reported to caused teratogenic effects in pregnant women.	(Rafii et al., 1997)
Oxaliplatin	Intestinal microbiota	The gut microbiota is involved in oxaliplatin-induced mechanical hyperalgesia, which was shown to be reduced in germ-free mice and in mice pretreated with antibiotics.	(Shen et al., 2017)
L−carnitine	Intestinal microbiota	L-carnitine is metabolized by the gut microbiota to trimethylamine (TMA) which is further metabolized in the liver to produce the toxic metabolite TMA-N-oxide (TMAO) that increases the risk of atherosclerosis and cardiovascular diseases.	(Koeth et al., 2013)

#### Gut Microbiome

As defined earlier, the gut microbiota, which is the largest microbial community in the human body, contains trillions of microorganisms (bacteria, archaea and micro-eukaryotes), including more than 1,000 different species of bacteria (Bacteroidetes and Firmicutes being the two major phyla in most cases), with more than 3 million genes. The gut microbiota is characteristic in each individual, and only one third of it is shared between different people. Studies on the gut microbiota constitute more than 80% of the overall publications on human microbiome (Cani, 2018), which reflects both the importance of its role in human health and the booming research in that area.

A classic case of microbiome-driven modulation of drug pharmacology and toxicity is the reduction of the cardiac glycoside, digoxin, by some strains of *Eggerthella lenta*, resulting in an altered metabolism, concentration and toxicity (Haiser et al., 2013). This differential reduction explains earlier reports of variability in digoxin metabolism between North Americans and southern Indians, who had variable abundance of the microorganism (Mathan et al., 1989). Another classic case is that of acetaminophen (**Table 2**), an analgesic, antipyretic and weak anti-inflammatory drug. Acetaminophen’s variable toxicity among individuals is due to variability in the amounts of p-cresol, produced by some gut bacteria, and which competes with acetaminophen’s o-sulfonation in the liver (Clayton et al., 2009). Current guidelines recommend assessing the microbiome activity before the administration of acetaminophen.

Ranitidine, an H2 Blocker, was found to be differentially degraded depending on the presence of gut bacteria with ability to cleave an N-oxide bond (Basit and Lacey, 2001). Insulin, an anabolic hormone and a peptide drug, was found to be degraded by some proteolytic enzymes produced by the gut microbiota, rendering it ineffective (Tozaki et al., 1997). Metformin, an anti-diabetic drug, was found to be more effective orally than parenterally because of gut-based pharmacological effects (Napolitano et al., 2014).

#### Skin Microbiome

The human skin is the largest body organ and it plays an essential role in the body’s natural immunity together with its associated microbial communities. The skin microbiota has more than 19 major phyla (Grice et al., 2009) and defends the human body against pathogens. These defense mechanisms include releasing antimicrobial peptides, maintaining pH and competing with pathogens for available nutritional resources.

Owing to its direct contact with the environment, the skin microbiota continuously interacts with, and thus adapts to, environmental xenobiotics. Studies have recently unveiled the role of topical xenobiotics on skin microbiota. For example, a study conducted on infants revealed that emollients resulted in decreased skin pH and increased bacterial richness, diversity, and abundance of *Streptococcus salivarius*, which seemed to improve atopic dermatitis treatment (Glatz et al., 2018). Chitosan is a natural biopolymer with immunomodulatory and antimicrobial properties. The inclusion of chitosan in coating textiles may reduce atopic dermatitis severity by modulating skin staphylococcal communities (Lopes et al., 2015). Additionally, topical treatment with coal tar in atopic dermatitis was found to decrease the abundance of *Staphylococcus aureus* and increase that of *Propionibacterium* on the skin, shifting the microbiome into a healthier state (Smits et al., 2019).

#### Urogenital Microbiome

The female urinary system is sterile in its normal state as a result of the urea content, which inhibits microbial growth, the mechanical flushing of pathogens by urine, and the lactic acid and hydrogen peroxide secreted by vaginal lactobacilli.

Like with other body sites, the urogenital microbiota mutually interacts with pharmaceutical xenobiotics. For example, tenofovir, an antiviral used in the treatment of HIV, was found to be metabolically depleted in African women by *Gardnerella vaginalis* and other anaerobic bacteria (Klatt et al., 2017). Combined oral contraceptives were shown to be associated with increased vaginal colonization by healthy lactobacilli, and with reduced bacterial vaginosis-associated taxa in another study (Brooks et al., 2017). Contraceptive rings were found to decrease the severity of bacterial vaginosis in African women by promoting the growth of vaginal lactobacilli and decreasing the abundance of *Gardnerella vaginalis* and *Atopobium vaginae* (Crucitti et al., 2018).

#### Oral Microbiome

The interaction between the oral microbiome and xenobiotics has long been studied as the microbial community of the oral cavity is constantly subjected to habits of eating, drinking and smoking that affect and change the oral microbiome on a daily basis.

In a recent study, silver, titanium dioxide, and iron nanoparticles were found to have the lowest inhibitory and antibiofilm concentration against dental caries-causing bacteria like *Streptococcus mutans* and *Streptococcus sanguinis*, when compared to other nanoparticle-containing solutions, antibiotics, and chlorhexidine (Lavaee et al., 2016). Hyaluronic acid treatment in peri-implantitis was found to decrease the severity of the disease by decreasing the abundance of colonizing bacteria (Soriano-Lerma et al., 2019). The antibacterial and antibiofilm activity of abietic acid on *Streptococcus mutans* growth was recognized as a potential protective measure against dental caries (Ito et al., 2019b). Of note, silver nanoparticles have complex interactions with mucosal and intestinal microbiomes (reviewed in Bi et al., 2020).

### Food Additives and Dietary Xenobiotics

Food is the source of energy and nutrients for the human body to grow, develop, and carry on everyday activities. The global human population is increasing over time, with incremental needs for ongoing food resources (Eshel et al., 2014). With this large demand, people have incorporated much more processed foods and artificial products into their diet to keep up with the rapid pace of their lifestyles. The enzymatic reserve of the gut microbiota has proven to play a pivotal role in metabolizing dietary compounds of natural, processed, or even artificial origins and making use of their metabolized products as a source of carbon or energy, not only for the microbiota itself, but also for the human host (Sharon et al., 2014).

Cooking or processing food may release undesirable xenobiotics that are not originally present in raw food such as polycyclic aromatic hydrocarbons, heterocyclic amines, and nitrosamines (Nogacka et al., 2019). The genotoxic consequences of the aforementioned xenobiotics are inextricable for both human and microbial genetic material (Nogacka et al., 2019). The gut microbiome may protect against the carcinogenic and genotoxic effects of these xenobiotics by biotransforming them to less toxic compounds or facilitating their excretion (Vanhaecke et al., 2008; Carmody and Turnbaugh, 2014). On the other hand, the gut microbiota may also transform heterocyclic amines, for example, into genotoxins, or may reverse the detoxification implied by host metabolism (Kassie et al., 2001). MeIQx (2-amino-3,8-dimethylimidazo[4,5-f]quinoxaline), one of the heterocyclic aromatic amines that are produced during red meat cooking, is believed to be associated with colorectal cancer. The gut microbiota is capable of transforming MelQx to MelQx-M1 which is of lower toxicity and mutagenicity to colon cells, thus it may be able to lessen the chances of cancer (Zhang et al., 2017a). The outcome of these diverse and sometimes contradictory effects will vary from person to person, and further studies in animal models and *in vitro* systems should address the effect of different microbial communities on a mixture of those chemicals.

On another front, the gut microbiome might be negatively affected by food additives, such as E171 or titanium dioxide. A recent study, conducted to observe the effect of E171 on the intactness of the colonic epithelium, showed that E171 altered the metabolites produced by the gut bacteria and helped forming a biofilm (Pinget et al., 2019). The imbalances in mucus layer thickness and epithelial permeability are involved in the development of colitis and colorectal cancer (Kim, 2014).

Non-caloric artificial sweeteners are currently consumed to give a sweet taste with much fewer or no calories. In one experiment, saccharin, sucralose, or aspartame were administered to mice in their drinking water. Consequently, mice developed marked glucose intolerance with distinct altered microbial composition (Suez et al., 2015). Xylitol, another food additive used to protect against caries, is capable of changing gut microbiota in mice, especially decreasing phylum Bacteroidetes, and enriching phylum Firmicutes and genus *Prevotella* (Uebanso et al., 2017). Benzoic acid is a preservative added to food to prevent bacterial and fungal growth. Dietary and supplement benzoic acid were proved to reduce *Lactobacillus* and *E. coli* in pig intestines (Mao et al., 2019). Emulsifiers, such as carboxymethylcellulose and polysorbate-80, caused microbial dysbiosis in mice and promoted low-grade inflammation which is one of the causes of metabolic syndrome (Chassaing et al., 2015).

### Environmental Chemicals and Pollutants

Environmental pollutants and chemicals have become an integral part of the surroundings of people. It is becoming necessary to include the microbiome in considering the safety and toxicity of these compounds, as microbial enzymes can alter their half-life and dynamics (Koppel et al., 2017). Melamine is used as raw material for manufacturing adhesives, paints, tables, and engineered wood. Melamine traces were found in soil and wastewater close to melamine factories (Wang et al., 2014a). In 2008, melamine toxicity led to the death of children because of gut microbial transformation of melamine to cyanuric acid. The latter form is an insoluble complex that precipitates in the kidney and causes kidney stones and is biotransformed by *Klebsiella terrigena*, together with *Pseudomonas* spp. strain A (NRRL B-12227), *Rhodococcus corallinus* (NRRL B15444R), *Klebsiella terrigena* DRS-1 (ATCC 700372), *Rhodococcus* sp. strain Mel, *Nocardioides* sp. strain ATD6, and the novel bacterium CY1 or *Melaminivora alkalimesophila* gen. nov., sp. nov (Zheng et al., 2013; Wang et al., 2014b).

Halogenated compounds are another class of chemicals used in agriculture as pesticides, pharmaceuticals, bleaching and disinfecting agents. They possess a negative impact on the gut microbiota, changing the Firmicutes-to-Bacteroidetes ratio to a dysbiotic one, and hence exposing humans to obesity or immune system perturbations. The gut microbiome is capable of nitroreductive metabolism and reductive dehalogenation for organohalogens (Atashgahi et al., 2018). One of these compounds, 2,4-D (2,4-dichlorophenoxyacetic acid), is one of the most commonly used herbicides around the world (Peterson et al., 2017). It disrupts hormonal pathways in plants and eventually leads to plant death. Hence, it is used to control weeds in agriculture and at homes. In a study to observe the effect of 2,4-D on mouse gut microbiota, a state of dysbiosis was observed with an occupational-dose exposure in 2,4-D-treated mice. Dysbiosis was not only on the taxonomic level—in terms of alpha diversity and richness, but it was also strongly manifested on the microbial metabolic pathways and metabolites. Among the microbial metabolic pathways affected was the metabolism of urea, amino acids, and carbohydrates (Tu et al., 2019).

Polychlorinated biphenyls (PCBs), albeit banned in 1977 for their toxic effects, are still present in the environment (Centers for Disease Control and Prevention (CDC), 2009). Exposure to PCBs is mainly through ingestion and may lead to impairment in the neurological functions and behavior especially in children (Rude et al., 2019). In an animal study, two groups of mice were exposed to PCBs, a wild type group and another with a mutation in RyR1 and FMR1 genes. PCBs caused a state of dysbiosis, increased inflammatory state, and a more permeable gastrointestinal barrier in the offspring especially in the group with genetic mutations. The latter group also was more predisposed to develop defects in neurobehavioral development (Rude et al., 2019).

Interestingly, the microbiome composition of a pregnant mother varies upon her exposure to PCBs, which also leads to a variation in the gut bacterial community of her fetus (Laue et al., 2019). In that case, the fetal exposure to PCB shifted the children’s microbiome towards order Bacillales and family Propionibacteriaceae, a shift that lasted up to eight years after their birth (Laue et al., 2019).

Another example highlighting the combined power of environmental exposure, individual genetic makeup, and gut microbiota dysbiosis on human health is the effect of organophosphate pesticide chlorpyrifos (CPF). In a mouse model with different apolipoprotein E (APOE) genotypes, in which CPF was administered after birth, a distinct taxonomical pattern was observed after exposure. APOE genotype and CPF exposure diversely affected the types of short-chain fatty acids in the brain (Guardia-Escote et al., 2020). A correlation had previously been established between gut and brain short-chain fatty acids, with observed impact on brain functionality (Sun et al., 2016).

Glyphosate is as a weedicide used around the globe (Green, 2018). Whether alone or in combination, glyphosate significantly changes the gut microbial niche through shifting the abundance of its two main phyla, Bacteroidetes and Firmicutes. Consequently, it affects brain plasticity, maternal behavior, and parenting (Dechartres et al., 2019).

Disruptions in microbial processing of amino acids and carbohydrates affect the balanced state of the host amino acids and carbohydrate metabolism, and may contribute to metabolic diseases, such as type 2 diabetes mellitus or obesity (Flint et al., 2008; Neis et al., 2015). On the other hand, microbial urease activity has been associated with diseases of the gastrointestinal tract such as ulcers or stomach cancer (Mora and Arioli, 2014).

Arsenic is a naturally present metalloid that poses its toxic effects on human health especially in its inorganic form. It is a potential cancer-causing chemical for lungs, kidneys, liver, skin, and bladder. It was proved to negatively affect cardiovascular, neurological, and immunological functions (Naujokas et al., 2013). An investigation of the role of the gut microbiota in arsenic metabolism, conducted in mice, showed that gut dysbiosis enhanced the toxic effects of arsenic, one way through increasing arsenic load and the other through promoting one-carbon metabolism. However, a balanced gut microbiota absorbs arsenic and promotes its methylation (Chi et al., 2019). In addition, dysbiosis may alter the expressions of some genes involved in p35 signaling and many genes involved in hepatocellular carcinoma development (Chi et al., 2019). While arsenic by itself may disturb the gut microbial community (Lu et al., 2014), *Bacteroides*, *Clostridium*, *Alistipes*, *Bilophila*, and other bacterial genera are able to methylate arsenic and can survive exposure to it owing to multiple arsenic resistance genes encoded in their genome (Yin et al., 2015; Yu et al., 2016).

Cadmium and lead are inorganic heavy metals that are not degraded and tend to accumulate in an ecological system (Daisley et al., 2019). They enter the human body through ingestion of contaminated food, water, and even particles cleared by the respiratory system (Satarug et al., 2003). Pesticides, fertilizers, corrosion of lead-containing plumbing, mining, lead batteries, and burning of coal are different sources of lead and cadmium pollution (Wuana and Okieimen, 2011; Li et al., 2015; Daisley et al., 2019). Human exposure to cadmium may cause hypertension, diabetes, myocardial infarction, and impaired kidney function, and may thus imbalance minerals homeostasis (Satarug et al., 2010). Lead can lead to neurotoxicities by affecting neurotransmitters in the nervous system and interfering with calcium in mitochondria, hence increasing oxidative stress and mitochondrial death (Mason et al., 2014). *Lactobacillus rhamnosus* GR-1 was shown to minimize human exposure by binding or sequestering heavy metals and consequently improving their excretion in feces (Daisley et al., 2019). As a result, lactobacilli are considered a safe cost-effective solution to decrease exposure to heavy metals and their severely negative potential on human health (Daisley et al., 2019).

Azo compounds are widely used in pharmaceutical, textile, food, cosmetics production. Azodyes may cause mutations in the human DNA, allergic reactions and cancer (Gičević et al., 2020). The gut microbiota expresses azoreductase enzymes to metabolize azodyes. Among azo-reducing bacteria are *Clostridium*, *Pseudomonas*, *Bacillus*, *Geobacillus*, *Lysinibacillus*, *Enterococcus*, *Eubacterium*, and *Escherichia* (Misal and Gawai, 2018). *Escherichia coli, Enterococcus faecalis, Enterococcus avium*, and *Bacillus cereus/thuriengensis* have been recently isolated from human stool samples with variable azoreduction rates (Zahran et al., 2019).

Triclocarban (3,4,4,9-trichlorocarbanilide, TCC) is an antibacterial ingredient in personal care products such as toothpaste and hand soap (Halden et al., 2017). Eventually, it integrates into the environment through wastewater as a persistent non degradable organic pollutant (Petrie et al., 2015). TCC was recently shown to shift the gut microbial composition to an increased Firmicutes and *Lactobacillus* taxa, and decreased Bacteroidetes in low-dose TCC-exposed rats. On the microbial metabolic level, TCC increased short-chain fatty acids concentrations suggesting that TCC enhanced bacterial fermentation. These changes play a role in the synthesis of fatty acids by the liver either directly or indirectly (Poole et al., 2016).

Triclosan (5-chloro-2-(2,4-dichlorophenoxy)phenol, TCS) is another broad-spectrum antimicrobial added to personal use products including cosmetics and toys (Halden, 2014; Poole et al., 2016). In a recent study on mice, TCS affected beneficial gut microbiota such as *Bifidobacterium*. TCS treatment also caused colonic inflammation in conventionally raised mice, supporting the evidence that gut microbiota plays a role in TCS-caused inflammation (Yang et al., 2018).

## Studying Toxicomicrobiomics

### Tools and Techniques

The tools for studying toxicomicrobiomics, as well as pharmacomicrobiomics, are no different from the general tools used to decode the microbiome at different levels: its taxonomic and genomic composition, gene content, functional potential, actual expression of its genes (at the RNA or protein level), and finally its actual function as reflected by the combined microbiome-derived metabolites. As such, microbiome profiling by amplicon sequencing, shotgun microbiome sequencing or metagenomics, metatranscriptomics, metaproteomics, as well as metabolomics and metabonomics are all valid tools for the analysis of xenobiotic-microbiome interactions (**Table 3**).

**Table 3:** Omics tools and technologies used in toxicomicrobiomic research.

Method	Main strategy	Questions answered	Limitations
**Amplicon sequencing (microbiome profiling)**	High-throughput sequencing of libraries of amplified taxonomic marker genes (16S or 18S rRNA subunit) to determine the microbiome composition by comparing sequencing read variants to taxonomic databases.	Who is there? What is the relative abundance of different taxa (operational taxonomic units/amplicon sequence variants)? Answers to these questions will help identify the community composition, alpha diversity, and beta diversity—if multiple samples are compared).	Cannot conclusively provide information about functional potential, since many pathways are not correlated with taxonomy, and many genes are horizontally transferred or species- and strain-specific. No guarantee that the detected bacteria are alive.
**Metagenomics**	Random sequencing of total DNA extracted from a microbial community/microbiome sample. The sequenced DNA includes fragments of different microbial and viral genomes, and possibly other soluble DNA, but the samples can be prefiltered or fractionated to separate cells from viral particles, or to get rid of soluble DNA prior to sequencing.	Who is there? What is the relative abundance of different taxa? Which non-cellular entities are there (e.g., viruses, bacteriophages, gene transfer agents)? What genes are there and what pathways are represented by these gene products? Novel organisms and previously unsequenced genomes may be identified by this method. The latter are typically known as metagenome-assembled genomes or MAGs.	Samples may contain host DNA that is hard to physically separate from microbial DNA, and that may even be hard to computationally remove. Like with amplicon sequencing data, the results cannot confirm that the detected taxa are living microbes. Additionally, genes detected are not necessarily expressed, and thus only provide information about functional potential—not actual functions.
**Metatranscriptomics**	Random sequencing of total RNA extracted from a microbial community/microbiome sample with the purpose of identifying genes that are actively transcribed (at the time of sampling) and quantifying their transcripts through mapping the sequence reads to complete genome sequences or sequence scaffolds.	The data may provide information on the community composition (taxa, abundance, and diversity), but the main questions answered by this technology are: Which genes have been transcribed (as a proxy for gene expression)? What are the relative abundances of these transcripts? Based on the expressed genes, which pathways or functional modules are engaged?	Like with all transcriptomic studies, not every RNA will be successfully translated into a protein, so the data may slightly overestimate gene expression. RNA is less stable than DNA, and different transcripts have different stability, thus there may be bias towards quantifying more stable transcripts. The method is highly sensitive to small changes that happen to the samples, from the time of collection till RNA extraction. This will thus lead to less reproducible results from similar or identical samples, and to spatially and temporally variable results within the same sample.
**Metaproteomics**	Determination of the sum of proteomes expressed by an entire microbial community at a given point of time and space using various mass spectrometric methods, most often coupled with chromatographic separation methods. The detected proteins may also include viral and, inescapably, host proteins. The resulting separated proteins or their fragments are detected through comparison of their masses to protein fragments in databases.	The method can provide information about the community composition (Who is there and how many)? and, in that regard, may be more accurate than gene-based methods as proteins may provide high-resolution strain identification. Additionally, and more importantly, the main questions answered by metaproteomics are: Which genes are being expressed into proteins? What is the differential abundance of these proteins? Which enzymes/pathways are functional? Which protein variants are produced? Sometimes information about posttranslational modifications are provided.	Metaproteomic data are harder to interpret than DNA and RNA-based data owing to the high complexity of proteins, and their higher chemical variability (in comparison to the rather homogeneous nature of nucleic acids). The method is highly sensitive to environmental contaminants and may thus have a considerable proportion of false-positive results. Like with metatranscriptomics, the sensitivity of the method may lead to lower reproducibility and higher spatio-temporal within-sample variability.
**Metabolomics and Metabonomics**	Metabonomics is a term coined from “metabolomics and (meta)genomics” and just refers to meta-metabolomic surveys, or the metabolites produced by the products of all genomes of a microbial community, as well as the host metabolites (Nicholson and Wilson, 2003). The term is often used interchangeably with metabolomics, since, after all, metabolomics measures all metabolites in a sample, and these metabolites will be coming from the host and its microbiome cloud.	Metabolomics is mostly concerned with the *actual* function that has been performed. Metabolites are thus like *‘smoking guns’*: they are the real evidence that an enzyme has produced its products, that a pathway was performed in a particular direction, and so on. Comparing metabolites to databases and using metabolomic profiles to classify samples provide answers about which different functional types or *metabotypes* are in the community. It is not surprising to find microbes with identical taxonomic assignments (or even near identical genomes) yet with drastically distinctive metabolomic profiles.	Differentiating host from microbial metabolites is challenging. Like metatranscriptomics and metaproteomics, this method is highly sensitive and consequently it may provide highly variable and less reproducible results because of the speed with which metabolites will change, and the spatial variations within a same sample (e.g., different parts of a stool samples will have highly variable metabolites). The sensitivity of the method also leads to noise and high vulnerability to minor contaminants, including those introduced during the process.

### Experimental Strategies for Discovery of Novel Xenobiotic-Microbiome Interactions

Using the above-mentioned ‘omics analysis technologies (**Table 3**), systematic studies need to be conducted to unearth new toxicomicrobiomic interactions. Strategies for these studies should benefit from environmental metagenomic approaches that seek to discover, for example, microbes and enzymes with biodegradation capabilities (Sayed et al., 2014; Bouhajja et al., 2016; Ngara and Zhang, 2018), or from pharmacomicrobiomic discovery methodologies (Bisanz et al., 2018). A remarkable example is the recent high-throughput systematic pharmacomicrobiomic screens to detect specific drug-bacterial interactions and separate them from drug-host interaction (e.g., Zimmermann et al., 2019a; Zimmermann et al., 2019b). Overall, experimental strategies, combining (wet) laboratory and computational (in silico approaches) may be classified into culture-based, sequencing-based, functional screening-based, and innovative hybrid strategies.

### Culture-Independent Sequencing-Based Methods

- *Amplicon sequencing:* Using amplicon sequencing to determine the microbiome profiles has been coupled to different in silico function prediction tools [e.g., PICRUST (Langille et al., 2013) and FishTaco (Manor and Borenstein, 2017)], although functional prediction from taxonomic markers remains of limited reliability (**Table 3**, limitations). Once a set of taxa have been predicted as xenobiotic metabolizers, they can be suggested as biomarkers and as targets for further culture-based or hybrid approaches (which could be systems-based or reductionist studies focusing on a representative of each taxon, or combinations thereof).
- *Shotgun metagenomics:* Shotgun sequencing provides the benefit of sequence-based discovery of enzymes and pathways [e.g., biosynthetic or biodegradation operons (Mohamed et al., 2013; Ziko et al., 2019)]. The discovery of cardiac glycoside-degradation genetic locus was mostly based on shotgun and metatranscriptomic sequence analysis (Haiser et al., 2013). This strategy has the advantage of gene-level analysis, which allows not only gene and pathway identification, but also the ability to determine closely related gene variants. However, like all DNA-based methods, there is no guarantee that sequenced genes are ever expressed in the environment from which they were sampled, which may mislead researchers. For example, a novel detoxifying enzyme-coding gene, sequenced from the oral microbiome, may suggest that its encoded enzyme acts at a certain pH or salt concentration, when the actual enzyme may never be expressed in the oral cavity, and thus may have no bioremediation value.
- *PCR libraries:* Here, instead of sequencing all metagenomic DNA, the DNA is amplified with primers (often degenerate) to amplify members of gene families that encode the desired protein (e.g., enzyme, resistance factor, efflux pump). The generated PCR product is then extracted, used to build a library (usually a plasmid library), propagated, then sequenced. This strategy helps determining a large number of possible variants of the desired gene family within the microbial community. This approach was used, for example, to discover and characterize novel thermostable mercuric reductases (Maged et al., 2019). As with all DNA-based approaches, the discovery of any valuable gene with this strategy is no guarantee that the gene is actually expressed in its cell’s natural habitat.

### Culture-Based Strategies

- Culturing the microbiome
  Advances are continuously being made to optimize various aerobic and anaerobic culture methods and technologies to grow members of the human microbiota, with emphasis on those that have been long considered as yet-uncultured or uncultivable (Forster et al., 2019; Ito et al., 2019a).
  Variations on this technology is the use of *in vitro* models for body sites or tissues, also considered as *ex vivo* models, e.g., fermentation chambers or reactors that simulate the human gut (Molly et al., 1993; Minekus et al., 1999; Takagi et al., 2016).
  An innovative technological breakthrough, which is a variation of the same concept of simulating body sites, is the iChip, developed to isolate bacteria from their natural habitats (Nichols et al., 2010; Berdy et al., 2017). It is possible, in the future, to see similar *in situ* technologies to isolate bacteria from human stool or human mucus/body fluids. These iChips may eventually evolve into smaller miniaturized, nano-scale, or robotic devices.
- *Systematic screens:* These are simple yet labor-intensive high-throughput screens, in which different resident bacteria (usually directly isolated from human body sites: gut, skin, vagina) are co-cultured with a set of xenobiotics (drugs, heavy metals, environmental toxins), and the chemical changes/modifications to these xenobiotics are detected and quantified *via* chromatographic separation followed by spectroscopic methods. A typical example is the recent impressive work of Zimmerman and coworkers (Zimmermann et al., 2019a).

All culture-based methods offer great advantage as they allow actual experimentation and screening; however, this strategy remains biased as it enriches our knowledge about many already well-studied organisms. Culture-independent technologies were specifically developed to overcome this type of knowledge bias.

### Function-Based (Functional Metagenomics Screening)

- In function-based metagenomic approaches, total DNA of environmental samples is extracted, fragmented, and expression libraries (in cosmids, fosmids, or other plasmids) are probed and screened for desired functions by positive or negative selection (Ngara and Zhang, 2018; Liu et al., 2019). These approaches offer the great advantage of actual expression of proteins, which allows testing their functionality, even if their producing microbes cannot be successfully cultured. For example, metagenomic libraries from soil, wastewater, river estuaries, or extreme environments (e.g., Red Sea brine pools (Mohamed et al., 2013; Sayed et al., 2014)) have been a good source for bioprospecting novel enzymes with ability to function at extreme conditions, or for the discovery of novel antibiotic or heavy-metal resistance genes *via* negative selection. This approach, which is well-established in applied and environmental microbiology, has been explored in screening the gut microbiome, for example, for resistance genes (Pehrsson et al., 2016) and biodegrading enzyme-coding genes (Cecchini et al., 2013). One limitation of this approach is the limited number of possible surrogate hosts (e.g., *E. coli*, *Lactococcus*, and yeast); however, anaerobic systems that are most relevant to the gut microbiota are being developed (e.g., in *Bacteroides* (Lam et al., 2018)).

### Hybrid Methods (Sequence/Culture Based)

- *Sequence-guided microbial augmentation:* Recently a composition-guided approach has been adopted to better define the structure and function of microbial communities at polluted environments. In an innovative step to precision bioremediation, Redfern et al. (2019) combined amplicon-based microbiome analysis and chemical profiling of polluted sites to improve the biodegradation capabilities of targeted microbial consortia. Microbial communities of environmental samples collected from several locations with different contamination levels were taxonomically classified by high-throughput sequencing. This novel framework relied on establishing relationships between chemicals and taxa to select candidates for biostimulation, bioaugmentation and genetic bioaugmentation. Specifically, the biostimulation target, *Geobacter*, documented as pollutant degrader, was detected at high abundance at all the sampling sites. The bioaugmentation targets comprised a K-strategist, *Mycobacterium*, and an r-strategist, *Thauera*, to guarantee rapid and long-term bacterial adaptation. The genetic bioaugmentation target, *Sphingomonas*, possessed the ability to receive catabolic plasmids from augmented donors (Redfern et al., 2019).
- *Reverse genomics:* Another quite recent approach is the use of reverse genomics to identify surface antigenic markers for hard-to-culture bacteria, which is a reverse vaccinology-inspired approach (Cross et al., 2019). These in silico-identified specific antigens are then cloned and expressed in heterologous systems, used to generate antibodies, which are then purified and deployed to capture the desired bacteria in their natural microbiome habitat, even at a single-cell level. Such microbial enrichment makes culturing the rarest strains possible (Cross et al., 2019).
- Finally, a hybrid approach that combines high-throughput systematic screens, functional metagenomics, and high-throughput sequencing was developed and adopted to discover plant polymer-decomposing enzymes (Nyyssonen et al., 2013). This approach, which integrates several of the above-mentioned strategies, uses multiplexed fluorometric and colorimetric assays to screen metagenomic libraries, and only sequences DNA inserts with promising desired phenotypes. The approach can easily be adapted to human microbiome samples, notably fecal samples, to discover xenobiotic-metabolizing enzymes, notably from organisms that cannot be cultured yet.

## Challenges and Future Aspects

Accruing reliable information about an individual’s microbiome may aid in preventing and treating certain diseases (Kosyakovsky, 2017). It may also lead to a decrease in the toxicity of some medications or the improvement in the efficacy of others (Haiser and Turnbaugh, 2012). A comprehensive “microbiome panel” may eventually be available as a routine laboratory screening to provide a microbiome profile of an individual and help defining the various options for a “healthy” microbiome. Once variants of healthy microbiomes and their biomarkers are defined, biomarkers for dysbiosis and disease susceptibility may also be defined. Consequently, xenobiotic-degrading bacterial taxa (or biomarkers thereof) may also be easily analyzed in the near future, and may be used in several therapeutic and toxicological decisions.

The highly disparate species and subspecies in the human microbiome interacting with every system in the body, including the immune system, raised the question about a core microbiome (Kuntz and Gilbert, 2017; Lloyd-Price et al., 2017). Core metabolic pathways are of more concern to comprehend the function and adaptation of the microbiome in a certain body location (Cardona et al., 2016). Researchers favor shotgun metagenomics to 16S rRNA gene profiling to analyze the whole genome of the microbial community (Cardona et al., 2016), which provides more information toward better deciphering of host-microbiome metabolic capabilities and interactions (**Table 3**). However, shotgun metagenomics is of higher cost and more complexity. In addition, achieving reliable personalized treatment based on pharmacomicrobiomics (and likewise toxicomicrobiomics) requires adopting standard reproducible approaches (Kashyap et al., 2017).

These challenges require multidisciplinary national and international collaborations to integrate big data, driven from multi-omics, to put pharmacomicrobiomics and toxicomicrobiomics into clinical applications. Microbiome studies should include populations with divergent geography and ethnicity (Lloyd-Price et al., 2017). Data and metadata of microbiome studies should be freely accessed over the time, according to peer-reviewed journal guidelines, to allow standardization and validation of bioinformatics and statistical analyses (Langille et al., 2018).

In this final section of the review, we propose ideas for practitioners, clinicians, and investigators in the field of microbiome research and its impact on human health.

### Open-Access Curated Databases

There is a scarcity of databases of ready curated knowledge for extraction by practitioners and investigators which may cost valuable time, resulting in hindering of the research or deterring researchers from conducting any investigation in the field. For this reason, building databases with such curated knowledge or helping already established databases as a volunteer team member, crowdsourcer or crowdfunder is a must for the advancement of the field. Specifically, linking toxicology databases, chemical databases, and drug banks to pharmacogenomic and pharmacomicrobiomic data resources is becoming a pressing need

### Predictive Models and Software Development

Building predictive models and developing software for simulating drug-microbiome interactions are essential as they will strongly reduce cost and researchers time. The only limitation to develop such models is the availability of curated knowledge, from which robust hypotheses can be driven and used for training and validating the model. Chemical similarity searches, if combined to gene/protein similarity searches, may allow the prediction of novel interactions between xenobiotic chemical moieties and different microbial enzymes.

### Microbiome Biomarkers as Future Non-Invasive Tools in Precision Medicine

As detailed above, the human microbiome is a rich source for discovery of biomarkers for diseases and adverse health outcomes. Using such biomarkers as non-invasive alternatives to biopsies and endoscopy, for example, would be a promising futuristic diagnostic (Ren et al., 2019) and predictive/preventive tool (Penalver Bernabe et al., 2018). For example, the human microbiota was found to release molecules and metabolites that affect the onset, progression, and treatment of cancer (Scott et al., 2019; Rao et al., 2020). True personalized cancer therapy may only be achieved through a full understanding of the effect of microbiome on chemotherapy (**Table 2**) and tumorigenesis, thus allowing proper patient stratification—based on specific biomarkers, microbiome types, and metabotypes (Rizkallah et al., 2010; Kuntz and Gilbert, 2017). Although inter-individual microbial diversity, emergence of novel and multiresistant microbial strains, and various microbial drug modification mechanisms may complicate or delay precision medicine, it remains the right choice for future theranotstics.

### Clear Guidelines for Clinical Practice

Hopefully, the availability of online resources, biomarker discovery, development of assays, models, and software, and conducting more research will help practitioners and investigators to develop novel guidelines for clinical practice, and to implement guidelines for some of the well-established xenobiotic-microbe and xenobiotic-microbial enzyme interactions.

## Author Contributions

RA conceived and conceptualized the article. All authors collected literature. NA and AR screened literature, made the final selection of reviewed articles, and drafted the article. ME reviewed toxicomicrobiomic strategies and tools. RA revised the draft and wrote the article in its final format. All authors read and approved the final format.

## Funding

RA and ME are funded by the Egyptian Academy for Scientific Research and Technology (ASRT) JESOR program (project # 3046); however, the funders had no input in the content of this article.

## Conflict of Interest

The authors declare that the research was conducted in the absence of any commercial or financial relationships that could be construed as a potential conflict of interest.
